# Comparison of palpation-versus ultrasound-guided fine-needle aspiration biopsies in the evaluation of thyroid nodules

**DOI:** 10.1186/1756-0500-1-12

**Published:** 2008-05-15

**Authors:** Ahmet Selcuk Can, Kamil Peker

**Affiliations:** 1 Division of Endocrinology and Metabolism, Department of Medicine, Vehbi Koc Foundation American Hospital, Sisli, Istanbul, Turkey; 2 Department of Pathology, Vehbi Koc Foundation American Hospital and Istanbul Pathology Group, Sisli, Istanbul, Turkey

## Abstract

**Background:**

The aim of this study was to compare the results of palpation-versus ultrasound-guided thyroid fine-needle aspiration (FNA) biopsies.

**Findings:**

Clinical data, cytology and histopathology results were retrospectively analyzed on all patients who underwent thyroid FNA biopsy in our outpatient endocrinology clinic between January 1998 and April 2003. The same investigators performed all thyroid FNAs (ASC) and cytological evaluations (KP). Subjects in the ultrasound-guided group were older, otherwise there were no differences in baseline characteristics (gender, thyroid function, the frequency of multinodular goiter, nodule diameter and nodule location) between groups. Cytology results in nodules aspirated by palpation (n = 202) versus ultrasound guidance (n = 184) were as follows: malignant 2.0% versus 2.7% (p = 0.74), benign 69.8% versus 79.9% (p = 0.02), indeterminate 1.0% versus 4.9% (p = 0.02), inadequate 27.2% versus 12.5% (p < 0.01). Malignant results were compared with Fisher's exact test. Other cytology categories were compared with chi-square test. Eighteen patients from the palpation- and 23 from ultrasound-guided group underwent surgery. In the palpation-guided group, the sensitivity of FNA was 100%, specificity 94%, positive predictive value 67% and negative predictive value 100%. In the ultrasound-guided group, the sensitivity of FNA was 100%, specificity 80%, positive predictive value 73% and negative predictive value 100%.

**Conclusion:**

We demonstrate that ultrasound guidance for thyroid FNA significantly decreases inadequate for evaluation category. We also confirm the high sensitivity and specificity of thyroid FNA biopsy in the diagnosis of thyroid cancer. Where available, we recommend universal application of ultrasound guidance for thyroid FNA biopsy as a standard component of this diagnostic technique.

## Findings

Thyroid nodules are commonly encountered in clinical practice. There are only two recent practice guidelines for the management of thyroid nodules, both published in 2006. American Association of Clinical Endocrinologists (AACE) recommends ultrasound (USG)-guided fine-needle aspiration (FNA), universally for all thyroid nodules that are ≥ 10 mm in diameter in euthyroid subjects [1]. In contrast, American Thyroid Association (ATA) recommends either palpation- or ultrasound-guided FNA biopsy [2]. As established guidelines differ on the utility of ultrasound guidance, we aimed to compare the results of palpation-guided and ultrasound-guided thyroid fine-needle aspiration biopsies in our clinical case series.

## Methods

We prospectively recorded the clinical information and cytology results of all consecutive patients who underwent FNA biopsy of thyroid nodules in the outpatient endocrinology clinic of our hospital in a computerized database. This prospectively-maintained database was retrospectively analyzed. All patients were examined by ASC, an endocrinologist who also performed all thyroid FNA biopsies between January 1998 and April 2003. Thyroid function tests and thyroid ultrasonography were routinely obtained. Free thyroxine (kit: FT4), free triiodothyronine (kit: FT3) and TSH (kit: TSH) were measured with electrochemiluminescence immunoassay in an Elecsys autoanalyzer. Kits and the autoanalyzer were supplied by the Turkish distributor of Roche Diagnostics, Indianapolis, IN, USA. Subjects were classified into euthyroid, hypothyroid and hyperthyroid categories according to the results of thyroid function tests. Thyroid scintigraphy with Technetium-99m was routinely obtained for all subjects who had low TSH levels and in some euthyroid subjects. Five patients had hyperactive nodules in thyroid scintigraphy and were excluded from this analysis. There were 299 subjects with thyroid nodules. Two hundred and twenty patients had a solitary thyroid nodule and 79 patients had a multinodular goiter. An ultrasound system (Ultramark 4+, Advanced Technology Laboratories, Washington, USA) with a 7.5 MHz linear array transducer was acquired by our outpatient endocrinology clinic in August 1999 and was used for all USG-guided biopsies since then. Seventy percent of palpation-guided biopsies were performed between January 1998 and August 1999 and 30% (n = 60) afterwards, at times when ultrasound system was temporarily unavailable. A published standard technique was used for palpation-guided FNA biopsies, performed by the investigator ASC [3]. All of the ultrasound-guided biopsies were performed between August 1999 and April 2003. The same investigator (ASC) performed all USG-guided biopsies with 22 or 26 G needles according to a previously published technique [4]. Local anesthesia with 2% lidocaine was routinely administered for both palpation- and USG-guided FNAs. Two needle aspirations were carried out for each nodule. If the material was judged macroscopically insufficient, up to four aspirations were performed to obtain additional material. On-site microscopic adequacy was not evaluated. Half of the slides were air-dried and stained with May-Grünwald-Giemsa and the other half were alcohol-fixed and stained with Hematoxylene-Eosine or Papanicolaou. The same investigator (KP) has evaluated all cytological smears. Cytological diagnoses were classified as recommended by existing guidelines into malignant, benign, indeterminate and inadequate for evaluation categories [2,3]. Six clusters of benign cells in at least two slides constituted adequate material for cytological diagnosis. Each cluster was composed of at least 15 cells. The smears that do not meet these criteria were assigned into inadequate category. Indeterminate samples included a pattern of follicular or Hurthle cell neoplasm or aspects of atypia suggestive, but not conclusive of the presence of a malignant neoplasm [3].

Baseline characteristics and FNA results of ultrasound-guided and palpation-guided groups were compared. Parametric variables were presented as mean ± standard deviation and Student's *t *test was used for comparison. Because of its positively skewed distribution, logarithmic transformation of nodule diameter was used in Student's *t *test. Categorical variables were presented as percentages and chi-square test was used for comparison. Because the expected frequencies of malignant results were less than five in both groups, each cytology category in palpation- and USG-guided groups was compared by chi-square test after collapsing the rest of the categories. Fisher's exact test was employed when the expected frequencies were less than five. Malignant and indeterminate FNA cytology results were categorized into positive tests in construction of 2 × 2 tables in the calculation of sensitivity and specificity, as surgery is recommended to patients with such results. Benign and inadequate cytology results were categorized into negative tests, as surgery is not recommended in these circumstances. In 2 × 2 tables, benign and malignant surgical histopathology results were categorized as negative and positive disease, respectively. The study was approved by the Scientific Research Review Board of Vehbi Koc Foundation American Hospital. As this analysis was based on a clinical case series and the subjects were patients who seek medical care, the informed consent was for the performance of thyroid FNA biopsy. Informed consent to perform thyroid FNA was obtained from all subjects in accordance with the hospital bylaws and Turkish Ministry of Health rules. The study was in compliance with the Helsinki Declaration.

## Results

In the palpation group, 126 patients had a solitary nodule, 35 patients had two nodules and 2 patients had three nodules, hence 202 FNAs were performed by palpation. In the ultrasound-guided group, 94 patients had a solitary nodule, 36 patients had two nodules and 6 patients had three nodules, hence 184 FNAs were performed with ultrasound-guidance. Subjects in the ultrasound-guided group were older, otherwise there were no differences in baseline characteristics (gender, thyroid function, the frequency of MNG, nodule diameter and nodule location) between the palpation- and ultrasound-guided groups, as shown in Table 1 and 2. In both palpation-guided and USG-guided hyperthyroid subgroups, four subjects had Graves' disease and three subjects had multinodular goiters. All hyperthyroid subjects had hypoactive nodules on thyroid scintigraphy. In the palpation group 4 (2%) biopsies were malignant, 141 (70%) benign and 2 (1%) indeterminate. In the ultrasound-guided group 5 (3%) biopsies were malignant, 147 (80%) benign and 9 (5%) indeterminate. The rate of malignancy was similar whether or not fine-needle aspiration biopsy was performed manually or under ultrasound guidance. Significantly more patients had benign and indeterminate results in the USG-guided group. Twenty-seven percent (55/202) of palpation-guided and 13% (23/184) of ultrasound-guided biopsies were inadequate for evaluation (χ 2:12.95, df: 1, p = 0.0003). Eighteen subjects from the palpation- and 23 subjects from the ultrasound-guided group underwent thyroid surgery. In the palpation group, histopathology showed two papillary thyroid carcinomas which were also malignant in prior cytology and were true positives. There were no false negative results in palpation-guided biopsies. There were 16 nodules that were benign in histopathology. Twelve of them were classified as benign, three as inadequate (a total of 15 true negatives) and one as indeterminate (false positive) in prior FNA biopsy. The false positive cytology result was found to be a Hurthle cell adenoma in surgical histopathology. In the palpation-guided group, the sensitivity of FNA was 100%, specificity 94%, positive predictive value 67% and negative predictive value 100%. In the ultrasound-guided group, histopathology after thyroidectomy showed seven papillary and one follicular thyroid cancers. From these, five nodules were malignant and three were indeterminate in prior cytology, so all of them were true positives. There were also no false negative results in ultrasound-guided biopsies. There were 15 nodules that were benign in final histopathology. Eleven of them were classified as benign and one as inadequate in prior FNA biopsy, so there were 12 true negatives. There were three indeterminate FNAs that were benign in final histopathology, two follicular adenomas and one adenomatous nodule and these were false positives. In the ultrasound-guided group, the sensitivity of FNA was 100%, specificity 80%, positive predictive value 73% and negative predictive value 100%.

**Table 1:** Comparison of baseline characteristics between subjects who underwent palpation-guided and ultrasound-guided thyroid fine-needle aspiration biopsies

	Palpation-guided	Ultrasound-guided	*P*-value
Subjects (*n*)	163	136	
Age (years)*	40 ± 12	44 ± 14	0.01
Men (%)†	23 (14%)	24 (18%)	0.40
Women (%)†	140 (86%)	112 (82%)	0.40
Euthyroid (%)†	151 (93%)	122 (90%)	0.61
Hypothyroid (%)†	5 (3%)	7 (5%)	0.61
Hyperthyroid (%)†	7 (4%)	7 (5%)	0.61
Solitary nodule (%)†	126 (77%)	94 (69%)	0.11
MNG (%)†‡	37 (23%)	42 (31%)	0.11

*mean and standard deviation are given and Students' *t *test is used in comparison †Categorical variables are expressed as percentages and chi-square test was used in comparison ‡MNG: multinodular goiter

**Table 2:** Comparison of nodule diameter, location and cytology between palpation-guided and ultrasound-guided thyroid fine-needle aspiration biopsies

	Palpation-guided	Ultrasound-guided	*P*-value
Nodule number (*n*)	202	184	
Log(nodule diameter in mm)*	1.22 ± 0.16	1.22 ± 0.21	0.71
Right thyroid lobe (%)†	95 (47%)	76 (41%)	0.08
Left thyroid lobe (%)†	81 (40%)	93 (51%)	0.08
Isthmus (%)†	26 (13%)	15 (8%)	0.08
Malignant (%)‡	4 (2.0%)	5 (2.7%)	0.74
Benign (%)†	141 (69.8%)	147 (79.9%)	0.02
Indeterminate (%)†	2 (1.0%)	9 (4.9%)	0.02
Inadequate (%)†	55 (27.2%)	23 (12.5%)	0.0003

*mean and standard deviation are given and Students' *t *test is used in comparison †Categorical variables are expressed as percentages and chi-square test was used in comparison ‡Fisher's exact test was used in comparison

## Discussion

Fine-needle aspiration biopsy is an essential diagnostic tool in the management of thyroid nodules. FNA can distinguish benign from malignant nodules and this has resulted in a large decrease in the number of patients undergoing surgery for excision of benign thyroid nodules and increased the cancer yield at surgery [1,2,5]. It is reported that 9 to 47% of palpation-guided and 4 to 21% of ultrasound-guided FNA smears are inadequate [6-13]. Hatada and coworkers reported that for nodules less than 2 cm in diameter, the sensitivity and accuracy of USG-guided FNA biopsies are significantly better than manual FNA [12]. Mehrotra and coworkers reported an unsatisfactory specimen rate of 46.8% for palpation-guided FNAs and 15.6% for USG-guided core-cutting needle aspirations [8]. From a large study with 9683 subjects, Danese and coworkers reported the sensitivity, specificity and accuracy of palpation-guided thyroid FNAs as 91.8%, 68.8% and 70.9% and of USG-guided FNAs as 97.1%, 70.9% and 75.6%, respectively [11]. The studies that compared palpation- and ultrasound-guided thyroid FNA biopsies were illustrated in table 3. There are wide differences in the rate of inadequate smears between studies. These differences could be attributed to the difference in the experience of the aspirating physician and the cytopathologist, and in the definition of an inadequate specimen among studies. A successful thyroid FNA requires an adequate specimen, high-quality smear preparation, and experience on the part of the both aspirator and cytopathologist [4]. In our study, all of the FNA biopsies were performed by the same endocrinologist (ASC) and all of the smears were read by the same pathologist (KP). Some previous studies that compared palpation- and ultrasound-guided FNAs included fewer number of nodules than our study [7,8,10,12]. In other previous studies, either the cytology or the aspiration were performed by different investigators [5,8,9,11,13]. In our study, the allocation to palpation- and ultrasound-guided groups was not random and was based on a certain time period, constituting a limitation for the interpretation of results. Ideally, comparison of different diagnostic methods should be performed on the same subject, at the same time, by the same aspirating physician, and the same cytopathologist should interpret all smears, as conducted in a prospective study by Cesur et al [7]. That study proved the superiority of ultrasound-guided thyroid FNA biopsies over palpation-guided FNA biopsies [7]. In clinical practice, it is recommended that ultrasound guidance should be sought after a failed manual thyroid FNA, in small nodules (less than 15 mm in diameter), in nonpalpable nodules, in lesions that are located in difficult-to-access locations, in nodules with extensive cystic change, fibrosis or calcification [3,6,11,14]. Ultrasound guidance is also helpful in directing the needle to solid portions of the cystic or mixed nodules and reduces the need for repeat FNAs [14]. Our results indicate that ultrasound-guided thyroid FNA cytology provides fewer unsatisfactory samples than palpation-guided thyroid FNAs. A decrease in the inadequate specimen rate is anticipated to improve the diagnostic accuracy of thyroid FNA biopsy. Ghofrani and coworkers reported that on-site adequacy assessment reduced the rate of inadequate smears from 12% to 7% in palpation-guided; from 7% to 5% in ultrasound-guided biopsies [9]. We did not have on-site assessment of FNA biopsies, a process that may have improved our diagnostic yield. In a recent study, Orija et al. reported the prevalence of thyroid cancer as 8.5% in patients with unsatisfactory FNAs and recommended the performance of at least two repeat FNA biopsies with ultrasound-guidance for initial inadequate smears [15]. As summarized in table 3, all published studies that compare palpation-guided with USG-guided FNAs show the superiority of the latter. Therefore, practice guidelines should universally recommend ultrasound-guided FNA biopsies in the management of thyroid nodules. Further studies should analyze cost-effectiveness of palpation-versus USG-guided thyroid FNAs.

**Table 3:** Inadequate biopsy rates in studies that compared palpation-guided versus ultrasound-guided thyroid fine-needle aspiration biopsies

Author and year	No. of nodules	Palpation-guided	Ultrasound-guided	Reference
Danese 1998	9683	9%	4%	[11]
Hatada 1998	166	30%	17%	[12]
Carmeci 1998	497	16%	7%	[5]
Mehrotra 2005	262	47%	16%	[8]
Ghofrani 2006	1502	17%	7%	[9]
Cai 2006	434	13%	6%	[13]
Cesur 2006	285	32%	21%	[7]
Izquierdo 2006	376	11%	7%	[10]
Can 2008	386	27%	13%	This study

## Conclusion

We have demonstrated that utilization of ultrasound guidance for thyroid FNA biopsies significantly decreases inadequate for evaluation category. We also confirm the high sensitivity and specificity of thyroid FNA biopsy in the diagnosis of thyroid cancer. In view of our results, where available, we recommend universal application of ultrasound guidance for thyroid fine-needle aspiration biopsy, as a standard component of this diagnostic technique.

## Competing interests

The authors declare that they have no competing interests.

## Authors' contributions

ASC performed thyroid fine-needle aspiration biopsies, collected and analyzed the data and drafted the manuscript. KP carried out cytological interpretation of thyroid fine-needle aspiration biopsies. All authors read and approved the final manuscript.
